# The time-resolved and extreme conditions XAS (TEXAS) facility at the European Synchrotron Radiation Facility: the general-purpose EXAFS bending-magnet beamline BM23

**DOI:** 10.1107/S1600577515017786

**Published:** 2015-10-17

**Authors:** O. Mathon, A. Beteva, J. Borrel, D. Bugnazet, S. Gatla, R. Hino, I. Kantor, T. Mairs, M. Munoz, S. Pasternak, F. Perrin, S. Pascarelli

**Affiliations:** a European Synchrotron Radiation Facility, CS 40220, 38043 Grenoble Cedex 9, France; b Institut des Sciences de la Terre, Université Joseph Fourier, 1381 rue de la Piscine, BP 53, 38041 Grenoble Cedex 9, France

**Keywords:** XANES, EXAFS, XAS beamline, microXAS, ESRF

## Abstract

BM23 is the general-purpose EXAFS bending-magnet beamline at the ESRF, replacing the former BM29 beamline in the framework of the ESRF upgrade. Its mission is to serve the whole XAS user community by providing access to a basic service in addition to the many specialized instruments available at the ESRF. BM23 offers high signal-to-noise ratio EXAFS in a large energy range (5–75 keV), continuous energy scanning for quick-EXAFS on the second timescale and a micro-XAS station delivering a spot size of 4 µm × 4 µm FWHM. It is a user-friendly facility featuring a high degree of automation, online EXAFS data reduction and a flexible sample environment.

## Abstract 2

BM23 is the general-purpose EXAFS bending-magnet beamline at the ESRF, replacing the former BM29 beamline in the framework of the ESRF upgrade. Its mission is to serve the whole XAS user community by providing access to a basic service in addition to the many specialized instruments available at the ESRF. BM23 offers high-signal-to-noise ratio EXAFS in a large energy range (5–75 keV), continuous energy scanning for quick-EXAFS on the second timescale and a micro-XAS station delivering a spot size of 4 µm × 4 µm FWHM.

## 1. Introduction   

A standard general-purpose EXAFS beamline on a bending-magnet source is an indispensable facility that the European Synchrotron has always offered to its user community. Until 2010, this role was played by beamline BM29 (Goulon *et al.*, 1995 ▸; Filipponi *et al.*, 2000 ▸). Within the framework of the ESRF upgrade, the experimental floor plan was modified to host new state-of-the-art beamlines: some beamlines were phased out and some were moved to new ports. Maintaining a standard bending-magnet EXAFS facility within the beamline portfolio allowed the ESRF to propose to the user community a coherent strategy for X-ray absorption spectroscopy, by providing access to a basic service in addition to cutting-edge techniques on specialized instruments: soft and hard polarized X-ray spectroscopy, micro- and nano-spectroscopy, high-energy-resolution absorption and emission spectroscopy, resonant inelastic X-ray scattering, energy-dispersive XAS, *etc*.

Within the new beamline portfolio, the bending-magnet general-purpose EXAFS beamline was integrated, with the energy-dispersive XAS beamline ID24, into the Upgrade BeamLine project 11 (UPBL11) dedicated to Time-resolved and Extreme conditions XAS (TEXAS project). At the same time, port BM29 was allocated to the upgraded protein crystallography project UPBL10. For this reason, the EXAFS facility on BM29 was closed in the summer of 2010 and transferred to port BM23, adjacent to beamline ID24. The new BM23 beamline accepted its first users in the spring of 2011.

The key missions of BM23 are to provide high signal-to-noise (S/N) EXAFS in a large energy range (5–75 keV), with a high degree of automation, featuring online EXAFS data reduction and a flexible sample environment. As the former BM29, BM23 has a simple optical scheme: a double-crystal fixed-exit monochromator, followed by a double-mirror harmonic rejection and mildly focusing device. The optics cabin provides monochromatic radiation to a single experimental station hosting standard XAS sample environments and detectors. The transfer of the beamline from port BM29 to port BM23 offered the opportunity for a major refurbishment, such as the implementation of granite supports for the monochromator and for the experimental bench, and the upgrade of the monochromator cooling system and crystal cage. Also, continuous scanning, as opposed to step-by-step scanning, was implemented and will eventually become the default data acquisition mode, leading to the possibility of recording full EXAFS in a few seconds. Finally, a micro-XAS station was made available to the user community, providing a focal spot of ∼4 µm × 4 µm in the energy range 5–40 keV, with an increase in brilliance varying from two to three orders of magnitude depending on the energy. The implementation of the latter two experimental facilities (quick-EXAFS and micro-XAS) provides an important bridge combining the time-resolved and extreme-conditions activities on BM23 and ID24. In this paper, we describe the present performance of BM23. §2 contains a brief description of the source, the optics, the experimental station and the detection systems. In §3, we describe the different sample environments that are made available to the user community. Finally, §4 highlights recent research results from the beamline.

## 2. Beamline overview   

The BM23 source is located on a standard ESRF bending magnet with *B* = 0.85 T. The source size is σ_*z*_ = 36.9 µm r.m.s. and σ_*y*_ = 126 µm r.m.s. in the vertical and horizontal directions, respectively. The beamline is aligned on the −9 mrad direction and accepts, through the primary slits located at 23 m from the source, a maximum divergence of 25 µrad in the vertical and 0.5 mrad in the horizontal directions. This leads to a maximum flux of 1.6 × 10^12^ photons s^−1^ (0.1% bandwidth^−1^) at the ESRF critical energy (21 keV). The beamline vacuum is separated from the accelerator vacuum by a 0.5 mm-thick Be window.

The first optical element is a double-crystal fixed-exit double-cam-type monochromator manufactured by Kohzu (Japan). The two crystals are in (+,−) geometry and diffract in the vertical plane. An angular range from 4.5 to 35° is accessible. The crystals are mounted on a single rotating plate. The rotation axis lies on the surface of the second crystal. The first crystal is mounted on a double-cam system which maintains a constant height for the exit beam during Bragg rotation. The monochromator has been refurbished with two objectives:

(*a*) To increase efficiency in the use of beam time, the design of the crystal support within the monochromator vacuum vessel was modified to permanently host three pairs of Si crystals (111, 311, 511) leading to an accessible energy range of 5 to 75 keV. The beamline setup, including crystal change, mirror and detector setup is performed in less than 1 h.

(*b*) To increase stability and reduce vibrations, the original metallic support has been replaced by a granite support and the monochromator cooling method was upgraded from the previous closed-loop cryogenic helium gas circuit to a liquid-nitrogen circulator. These modifications led to a decrease of the vibration level measured on the first crystal surface from 2.5 µrad r.m.s. down to 54 nrad r.m.s.

The vertical primary slits located before the monochromator define the vertical divergence of the beam and, together with the Darwin width of the crystals, the energy resolution Δ*E*/*E* of the monochromator. The divergence of the beam and the choice of the crystal pair are adjusted at each energy to obtain an energy resolution Δ*E*/*E* smaller or equal to Δ*E*_ch_/*E*, where Δ*E*_ch_ is the core hole lifetime broadening. The monochromator main Bragg rotation resolution (0.5 µrad) allows suitable sampling for all accessible edges. The energy reproducibility of the monochromator is equal to 20 meV FWHM at the Cu *K*-edge.

The second optical element, located after the monochromator, is a double-mirror system for harmonic rejection. It is based on total reflection through a double X-ray mirror configuration with an incidence angle variable from 2 to 5 mrad on three different stripes: Si, Pt and Rh. The first mirror lies flat and deflects the X-ray beam downwards. The second mirror deflects the X-ray beam upwards. The system allows harmonic rejection to better than the 10^−5^ level over the 5–40 keV energy range. The second mirror is bent in the meridional plane. The radius of curvature can be varied from ∞ (flat) to 1.95 km to allow modest vertical focusing of the beam at the different sample position. The small distance of 1.3 mm between the surfaces of the mirrors coupled with the small incidence angle offer fixed-exit operation when changing the angle from 2 to 5 mrad. During an EXAFS scan the system does not move.

**Figure 1:**
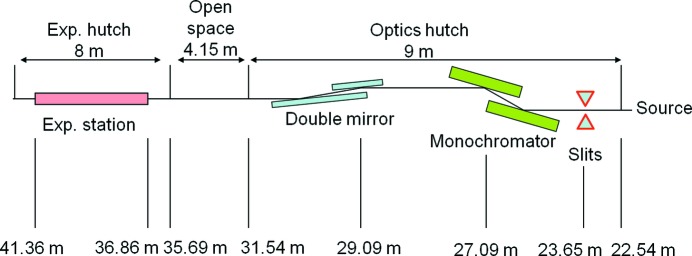
BM23 beamline: optical layout.

The principal characteristics of the BM23 beamline are listed in Table 1 ▸ and a schematic view is presented Fig. 1 ▸.

The experimental station is equipped with a large granite block of 1.2 m × 4.5 m, which is able to accommodate all kinds of experimental environments required by the user. It has been designed to avoid mechanical vibrations, to increase experimental space, to rationalize experiments installation and to facilitate access to services (power supply, ethernet connections, serial line and coaxial cables, motors supply and control, vacuum lines, He recovery and pumping system). The granite has shown remarkable stability, with no amplification of vibrations at frequencies lower than 50 Hz in the three (*x*, *y*, *z*) directions.

The beamline is optimized for transmission and fluorescence measurements. Three ionization chambers (Pettifer *et al.*, 1999 ▸) coupled to Novelec medium/high-sensitivity amplifiers (Gauthier *et al.*, 1995 ▸) allow transmission measurements of sample and reference simultaneously. The current amplifiers are equipped with an integral voltage-to-frequency converter which is optically coupled to the counting card. A 13-element Ge detector from Camberra is used for fluorescence measurements. The Ge detector is coupled to a DXP-xMAP readout electronic from XIA LLC. This configuration demonstrates linear operation up to a maximum input count rate of 80000 counts per second per channel. The beamline is also equipped with a Vortex Silicon Drift Detector (SDD) model 90EX (from Hitachi High-Technologies Science America, Inc.) with 1 mm-thick active material and CUBE pre-amplifiers (from XGlab). The readout is performed *via* a single-channel DXP Mercury module from XIA LLC. This configuration demonstrates linear operation up to a maximum input count rate of 500000 counts s^−1^. Angle-dispersive X-ray diffraction is also possible *via* a MAR 165 CCD detector permanently installed on the beamline, in combination with the XAS measurements.

In addition to the standard step-by-step acquisition mode, a continuous acquisition scheme is implemented for both transmission and fluorescence modes (Prestipino *et al.*, 2011 ▸). This acquisition mode will eventually become the default data acquisition mode and leads to the possibility of recording full EXAFS in a few seconds. It provides very complementary performance to that of ID24.

In addition to the room-temperature multi-sample holder, various sample environments are available on the beamline. He cryostats, developed at ESRF, offer a temperature range from 1.6 to 400 K. In these cryostats, the sample is coupled to the cold head *via* He gas, allowing reliable temperature measurements to be made independently of the sample thermal conductivity. Cryostats are compatible with transmission or fluorescence measurements. The sample chamber of the cryostats is shielded with ultrapure 1 mm-thick aluminium to allow fluorescence XAS measurements on diluted samples. The cryostats offer also the possibility of measuring liquid samples and are compatible with a reductive atmosphere.

Furnaces (Filipponi & Di Cicco, 1994 ▸) are available on the beamline and offer the possibility of performing high-temperature *in situ* studies of solids and liquids. The maximum temperature is ∼3000 K with high heating/cooling rate (100 K s^−1^) under vacuum. High-temperature reactors (Bellet *et al.*, 2003 ▸; Guilera *et al.*, 2009 ▸) are also available for *in situ* studies of catalytic processes. These cells can reproduce realistic three-way catalyst conditions on compressed pellets with temperatures up to 1300 K, in oxidizing/reducing gas atmosphere at high flux. Plug flow capillary microreactors (Figueroa *et al.*, 2013 ▸) are available for studies on catalytic beds in powder form, at temperatures up to 1200 K and in oxidizing/reducing atmosphere up to 20 bar. The reactors can be coupled to an *in situ* mass spectrometer. These devices are all compatible with transmission or fluorescence geometry.

A large-volume Paris–Edinburgh press (Besson *et al.*, 1992 ▸) allows XAS to be performed on mm^3^ samples at high pressure up to 15 GPa and temperatures up to 2000 K. The large volume available in this press is particularly important to adapt the thickness of the sample to XAS in transmission. This is important for studies on dilute systems. Thus, the Paris–Edinburgh press is particularly suited to geochemistry/geophysics applications related to the upper mantle or to the subduction zone. Angle-resolved X-ray diffraction can also be recorded before or after the XAS measurement and offers long-range-order information that complements the local structure determination using EXAFS. XRD is also often used to calibrate pressure and/or temperature by means of reference samples inserted close to the probed sample volume. In 2012 a new micro-XAS station was made available to the user community, with an increase in brilliance of a factor two to three orders of magnitude depending on the energy. The micro-XAS station is based on a set of Pt-coated mirrors in a Kirkpatrick–Baez (KB) geometry, a sample support, a microscope and detectors. The incident angle of the two mirrors can be varied from 2 to 8 mrad allowing XAS operation between 5 and 40 keV. The KB mirrors produce a round focal spot of less than 4 µm × 4 µm FWHM. The stability of the beam during an XAS energy scan is better than 1 µm keV^−1^ in both transverse directions. The micro-XAS station is equipped with mini ionization chambers developed in-house and is compatible with the beamline fluorescence detectors. The setup is complemented by a visible microscope with variable magnification. The choice of a relatively large focal distance (*q* = 0.5 m for the second mirror) is the result of a compromise between focal spot size, data quality and a large sample environment volume of about 10 cm × 10 cm × 10 cm for cryostats, high-pressure diamond anvil cells, ovens and alignment tools. The micro-XAS facility is also compatible with the MAR CCD camera mentioned above, enabling collection of XRD patterns.

## 3. Ancillary facilities   

Users on BM23 have access to several ancillary facilities.

(i) A user chemistry laboratory, equipped with all standard XAS sample preparation methods (balance, pellet press, crusher, ball milling machine, Millipore filters, Schlenk line, small chemistry items, furnaces, fume hood, basic chemistry products, *etc.*).

(ii) A second chemistry laboratory, dedicated to catalysis and chemistry equipped with a DRIFT spectrometer, a stopped-flow cell, a UV–VIS spectrometer and a mass spectrometer.

(iii) A preparation laboratory, dedicated to high-pressure activities.

(iv) A general-purpose mechanical workshop.

## 4. Facility access   

The beamline BM23 is accessible through the standard ESRF peer-reviewed proposal system; more information can be found at http://www.esrf.eu/UsersAndScience/UserGuide/Applying. Beam time can also be requested through the Long-Term Proposal procedure (http://www.esrf.eu/UsersAndScience/UserGuide/Applying/LongTermProjects). Finally, beam time can also be obtained on a commercial basis by contacting the Business and Development Office (http://www.esrf.eu/cms/live/live/en/sites/www/home/Industry.html).

## 5. Highlights   

The key mission of BM23 is to provide high-quality EXAFS in a large energy range (5–75 keV), with high S/N ratio up to large *k*-range, with a high degree of automation, featuring online EXAFS data reduction and a flexible sample environment. To illustrate the quality of the spectrometer, three examples are described in the following paragraphs. The first one describes an EXAFS study of negative thermal expansion in CdTe. It underlines the quality and the accuracy of the EXAFS measurements performed on the BM23 spectrometer in transmission mode. The second concerns *in situ* EXAFS characterization on supported Pt/CeO_2_ catalysts for CO oxidation. It illustrates the versatility of the beamline and the performance obtained in fluorescence mode. Finally the last example illustrates the possibility offered by the new micro-XAS station by combining XRF and XANES analysis on weathered peridotites from New Caledonia.

### 5.1. Negative thermal expansion in crystals with the zincblende structure: an EXAFS study of CdTe   

Thermal expansion is a well known effect of anharmonicity. In two-atom molecules, the asymmetry of the interaction potential energy gives rise to positive thermal expansion. In condensed systems, where the potential energy is defined in a many-dimensional configuration space, negative thermal expansion (NTE) can also be observed in relatively simple crystal structures; as a matter of fact, a number of tetrahedral semiconductors, such as Si, Ge, GaAs, CdTe and CuCl, undergo isotropic NTE within limited low-temperature intervals. The interest in NTE has been reawakened by the discovery of substances that undergo strong isotropic NTE over extended temperature intervals and are generally characterized by framework structures. The recent discoveries not only stimulated applied research into specifically tailored materials but also renewed the interest in a deeper basic understanding of the mechanisms at the origin of NTE. According to a model based on central force mechanisms, the net lattice expansion is the result of a competition between a positive bond-stretching contribution due to the anharmonicity of the pair potential and a negative contribution due to tension effects. When tension effects prevail over bond stretching, the solid contracts upon heating. Dilatometry and Bragg diffraction are sensitive only to the lattice thermal expansion, and cannot distinguish between bond-stretching and tension contributions. If this is the aim, one can exploit the complementary information on local structure and dynamics obtained from correlation-sensitive probes, such as EXAFS and total scattering.

**Figure 2:**
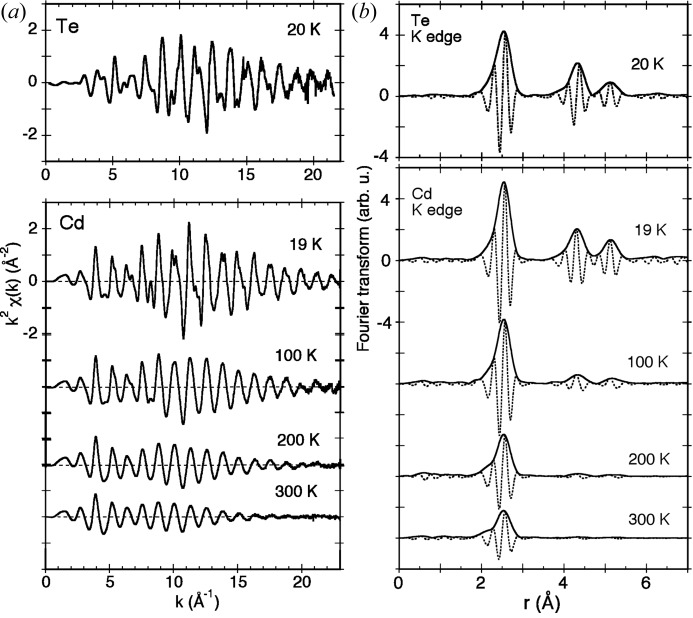
(*a*) EXAFS signals *k* ^2^χ(*k*) at the *K*-edge of Te in CdTe at 20 K (top panel) and at the *K*-edge of Cd at some selected temperatures (bottom panel). (*b*) Modulus (continuous lines) and imaginary parts (dotted lines) of the corresponding Fourier transforms given by Abd el All *et al.* (2012 ▸).

This experiment was aimed at performing a detailed investigation of the local origin of negative thermal expansion in CdTe. This work was part of an ongoing project devoted to the study of the vibrational properties and thermal disorder in zincblende crystals. In parallel, these studies are also aimed at searching for the ultimate limits of accuracy of EXAFS analysis (Abd el All *et al.*, 2013 ▸). EXAFS was measured at both the *K*-edges of cadmium and tellurium in CdTe, from liquid-helium to room temperature, in order to investigate the local thermodynamic behavior (Abd el All *et al.*, 2012 ▸). Fig. 2 ▸ reports some of the data measured at both edges. The temperature dependence of the structural parameters obtained from a separate analysis of the two edges was perfectly consistent. The positive contribution to the thermal expansion due to the bond stretching and the negative contribution due to the tension effects were disentangled and quantified in terms of the bond thermal expansion and the perpendicular mean square relative displacement. Comparison with previous EXAFS results for Ge and CuCl showed that relevant correlations can be established between a number of local parameters measured by means of EXAFS and the properties of the lattice NTE of tetrahedrally bonded semiconductors.

### 5.2. Supported Pt/CeO_2_ catalysts for CO oxidation   

Platinum group metals (PGM) supported on CeO_2_ are often used as active catalysts for oxidation of toxic carbon monoxide (CO). However, due to the economic, social and ecological costs of the PGMs, these metals have to be employed as efficiently as possible. Reducing the particle size to the nanoscale not only increases the efficiency but additional catalytic effects can emerge (nanoclusters, single-atom catalysis). In this context, two preparation methods were used to obtain catalysts characterized by different Pt-support interaction: (i) one-pot decomposition of a solution containing Pt(NO_3_)_2_ and Ce(NO_3_)_3_ was performed in order to achieve atomic dispersion of Pt in the CeO_2_ lattice, and (ii) impregnation of Pt(NO_3_)_2_ on high-surface-area cerium oxide was carried out to distribute the smaller noble metal particles on the surface.

**Figure 3:**
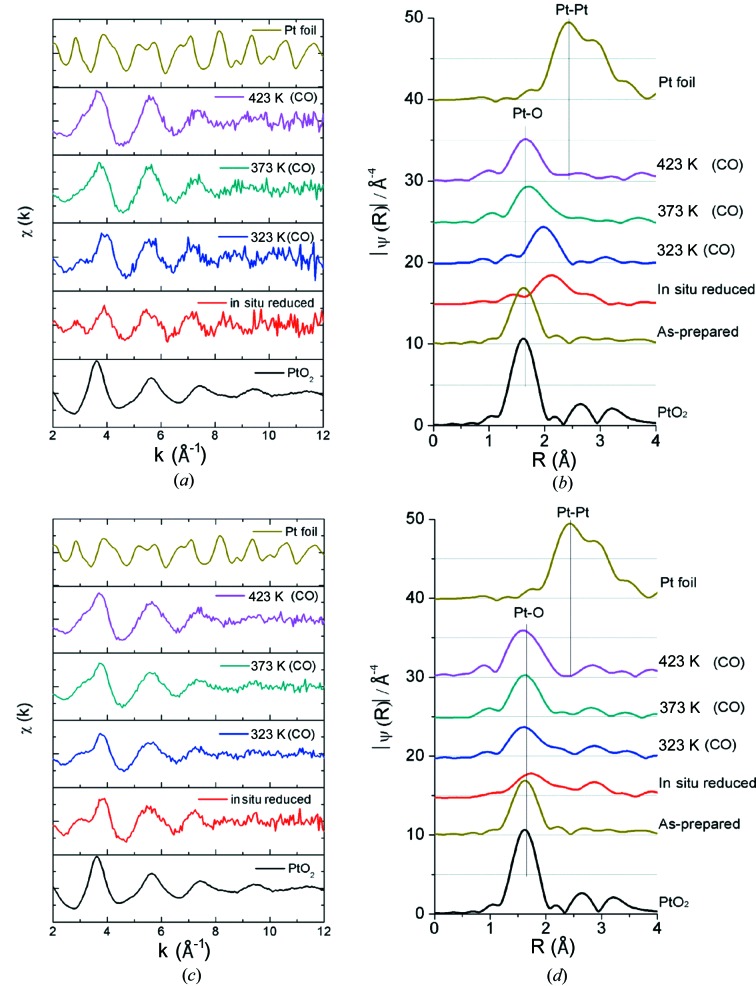
Fourier transform and EXAFS signals χ(*k*) at the *L*_3_-edge of Pt in (*a*, *b*) impregnated and (*c*, *d*) one-pot 2.5 wt% Pt/CeO_2_ catalysts during *in situ* reduction and CO oxidation at three different temperatures (323, 373 and 423 K). Fourier transformation of the *k* ^3^-weighted EXAFS function *k* ^3^χ(*k*) into *R* space using a Kaiser–Bessel window function was performed in the range 2–10 Å^−1^, yielding the function |χ(*R*)| (Å^−4^).

Interestingly, the oxygen storage capacity (OSC) of CeO_2_ in the one-pot sample is found to be superior to the impregnated catalyst OSC. In order to link the catalytic activity to the electronic and structural properties of the platinum and cerium species during reduction and CO oxidation, XAFS was coupled with mass spectrometry at BM23. We show here only the Pt *L*_3_ data which provide the most significant results. Due to the low Pt content (2.5 wt%), EXAFS spectra were recorded in fluorescence mode using the 13 element Ge array detector. The amplitude of the Fourier transforms and the EXAFS signals χ(*k*) at the *L*_3_-edge of Pt in the impregnated and in the one-pot 2.5 wt%/CeO_2_ catalysts recorded during *in situ* reduction in 5%H_2_/He and CO oxidation (2000 p.p.m.) at three different temperatures (323, 373 and 423 K) are presented in Fig. 3 ▸.

EXAFS analysis shows that the local environment of Pt in the two samples is very different. In the one-pot synthesized samples it is very similar to that in platinum oxide (only oxygen as nearest neighbours), whereas in the impregnated samples the local environment of platinum is composed of both oxygen and platinum atoms. In the latter, after *in situ* reduction, the Pt—O bond distance is considerably longer than that in PtO_2_ (2.10 ± 0.05 Å compared with 2.00 ± 0.01 Å in PtO_2_); see data at 323 K in Fig. 3 ▸. The observed structural differences between the two samples have been interpreted with different atomic models for the Pt–CeO_2_ interaction. These are characterized by an unequal availability of platinum for the catalytic reaction, thereby providing a link to the difference in CO oxidation activity of these systems (Gatla *et al.*, 2015 ▸).

### 5.3. Distribution of iron and nickel in the weathered peridotites from New Caledonia   

The New Caledonia nickel ore and derived metallurgical products represent about 30% of the world’s resources, with an annual production of more than 100000 tons. The New Caledonia ophiolite is the world’s largest outcrop of ultrabasic rocks, derived from the obduction of peridotites on the continental Norfolk ridge during the Eocene (∼34 My). Owing to tropical weathering, ophiolite is strongly altered due to the percolation of meteoric water (Ulrich, 2010 ▸).

Although the mineral paragenesis of ophiolite weathering has been relatively well documented for about 30 years, the weathering and concentration processes are still not well understood and could be potentially of great importance for both fundamental research and industrial productivity. This ongoing study (Muñoz, 2015 ▸) is aimed at characterizing both Ni and Fe (speciation and oxidation state) to gain information regarding the local oxygen fugacity associated with different Ni speciation observed along the weathering profile.

**Figure 4:**
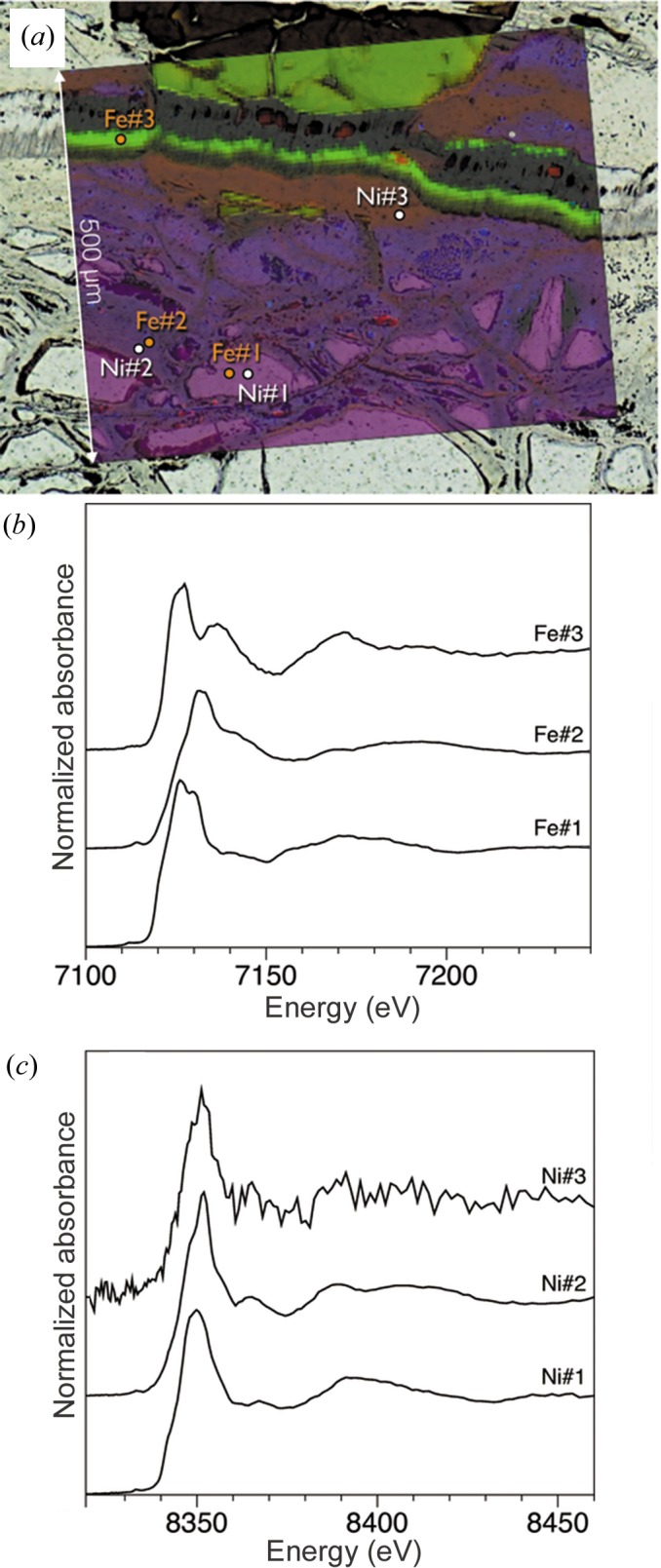
(*a*) Optical image and XRF RGB-map (red: Fe; green: Mn; blue: Ni) of a weathered peridotite from the New Caledonia ophiolite. Fe (*b*) and Ni (*c*) *K*-edge XANES of different regions of the rock, highlighting the presence of different types of minerals with contrasted redox states for iron, and variable amounts of nickel.

Fig. 4 ▸ shows two-dimensional XRF and µXANES analysis performed with the new micro-XAS facility on BM23. The remarkable beam stability allowed scanning of a wide energy range (beam movements are less than 2 µm for a 2000 eV range), so that both Fe and Ni *K*-edges could be scanned one after the other, at a unique position. Preliminary results shown in Fig. 4 ▸ are obtained on a rock prepared as a thin section from the rocky saprolite alteration level of the ophiolite. The XRF map is 500 µm × 670 µm in size and the pixel size is 5 µm × 5 µm. The red–green–blue coloured map is based on the respective intensity of the Fe, Mn and Ni *K*α lines. Various XANES spots are labelled according to the XANES spectra collected at the Fe and Ni *K*-edges [Figs. 4 ▸(*b*) and 4(*c*), respectively]. Spectra labelled #1 and #2 were collected in olivine and serpentine minerals. Fe#3 was collected in a carbonate, while Ni#3 corresponds to a Ni-depleted serpentine. These preliminary results highlight the presence of minerals with different redox states for Fe, and variable amounts of nickel in serpentine. Moreover, unexpected Fe-carbonates are observed in complex mineral veins close to the mesh texture.

## 6. Discussion and conclusion   

BM23 is the general-purpose EXAFS bending-magnet beamline at the ESRF, replacing the former BM29. Its mission is to serve the whole XAS user community by providing access to a basic service in addition to the many specialized instruments available at the ESRF. BM23 offers high S/N ratio EXAFS analysis in a large energy range (4–75 keV), continuous energy scanning for quick-EXAFS on the second timescale and a micro-XAS station delivering a spot size of 4 µm × 4 µm (FWHM). It is a user-friendly facility featuring a high degree of automation, online EXAFS data reduction and a flexible sample environment.

## Figures and Tables

**Table 1:** BM23 beamline: main characteristics

Beamline name	BM23	
Source	0.85 T ESRF bending magnet	
Primary slits	10 mm × 0.3 mm located at 23.5 m	
Monochromator	Fixed-exit double-crystal monochromator (DCM) Si(111/311/511)	
Mirrors	Double mirrors 2–5 mrad Si/Pt/Rh stripes	
Energy range	5−75 keV	
Beam size (unfocused)	15 mm by 1 mm	
Flux on sample (unfocused)	1.2 × 10^11^ photons s^−1^ at 21 keV in 0.35 mrad × 14 µrad at 200 mA using a Si(111) DCM and RH mirrors at 2 mrad†	
Beam size (focused)	4 µm × 4 µm (FWHM) using a pair of Pt-coated mirrors in KB geometry	
Flux (focused)	4 × 10^9^ photons s^−1^ at 21 keV in 0.02 mrad × 10 µrad at 200 mA using a Si(111) DCM and KB mirrors at 3 mrad†	
Detectors	Ionization chambers, diodes, 13-element Ge detectors, Si drift diodes, MAR 165 CCD	
Sample environments	Automatic sample changer, low-temperature (1.6–400 K), high-temperature (300–3000 K), high-pressure (1–100 GPa), high-temperature reactors, plug flow capillary microreactors	

† Measured with a calibrated Si PIN diode.
